# Network Analysis of Antimicrobial MIC Profiles Reveals Extensive Covariation in Chicken-Associated *Escherichia coli* from Large-Scale Breeding Flocks in Central Hungary

**DOI:** 10.3390/antibiotics15100999

**Published:** 2026-10-09

**Authors:** Ádám Kerek, Gergely Álmos Tornyos, Edit Szarka, Levente Hunor Husz, Máté Hetyésy, László Kovács, Ákos Jerzsele

**Affiliations:** 1 Department of Pharmacology and Toxicology, University of Veterinary Medicine Budapest, H-1078 Budapest, Hungary; tornyos.gergely.almos@student.univet.hu (G.Á.T.); szarka.edit@student.univet.hu (E.S.); husz.levente.hunor@student.univet.hu (L.H.H.); hetyesy.mate@student.univet.hu (M.H.); jerzsele.akos@univet.hu (Á.J.); 2 National Laboratory of Infectious Animal Diseases, Antimicrobial Resistance, Veterinary Public Health and Food Chain Safety, University of Veterinary Medicine Budapest, H-1078 Budapest, Hungary; kovacs.laszlo@univet.hu; 3 Department of Animal Hygiene, Herd Health and Mobile Clinic, University of Veterinary Medicine Budapest, H-1078 Budapest, Hungary

**Keywords:** antimicrobial resistance, antimicrobial stewardship, poultry, *Escherichia coli*, minimum inhibitory concentration, MIC-covariation network, Central Hungary, whole-genome sequencing

## Abstract

**Background:** The surveillance of non-clinical bacteria from food-producing animals can reveal antimicrobial selection patterns without conditioning sampling on clinical disease, but categorical resistance estimates are difficult to compare when organism-specific breakpoints are incomplete. We aimed to characterize antimicrobial MIC distributions in chicken-associated *Staphylococcus* spp., *Enterococcus* spp., and *Escherichia coli* from Central Hungary and to determine whether *E. coli* MIC profiles formed structured covariance networks. **Methods:** The dataset comprised 133 isolates collected in 2022–2023 (28 *Staphylococcus* spp., 59 *Enterococcus* spp., and 46 *E. coli*). MIC distributions were summarized using MIC_50_/MIC_90_, and *E. coli* MICs were analyzed by Spearman correlation with Benjamini–Hochberg correction. Historical threshold classification was retained only as a sensitivity analysis, and nine *E. coli* genomes provided contextual genomic information. **Results:** *Staphylococcus* spp. showed lower amoxicillin MIC_50_/MIC_90_ values (0.25/1 µg/mL) than *Enterococcus* spp. (1/256 µg/mL), illustrating marked cross-genus differences. In *E. coli*, MIC_50_/MIC_90_ values included 16/512 µg/mL for ceftriaxone, 128/256 µg/mL for neomycin, 32/128 µg/mL for enrofloxacin, and 32/>512 µg/mL for trimethoprim–sulfamethoxazole. Ten associations met |ρ| ≥ 0.40 and adjusted q < 0.05, forming two connected components that persisted after sensitivity analyses for upper-bound accumulation and the amoxicillin–amoxicillin/clavulanic acid relationship. The nine genomes showed heterogeneous resistance-associated gene calls. **Conclusions:** Continuous MIC analysis revealed phenotypic covariance not captured by categorical summaries alone and identified region-specific targets for stewardship-oriented follow-up. Inference remains isolate-level rather than farm-adjusted, and the genomic panel is not representative of the phenotypic cohort; longitudinal sampling linked to antimicrobial-use data and larger matched phenotype–genotype datasets are therefore warranted.

## 1. Introduction

Antimicrobial resistance (AMR) in food-producing animals sits at the intersection of animal health, public health, food production and environmental microbiology. Antimicrobial exposure can select resistant bacterial populations in livestock, while commensal organisms provide a practical record of that selection pressure even in the absence of overt bacterial disease [1,2]. For surveillance purposes, the value of these organisms lies less in any single isolate than in the distribution of susceptibility phenotypes across a defined population and in the persistence of recurring resistance structures over time.

Non-clinical indicator bacteria are particularly useful for One Health AMR surveillance because they characterize the background bacterial population rather than only organisms recovered from clinically affected animals. Such bacteria may also act as reservoirs of acquired resistance determinants that circulate among livestock, the food chain, humans, and the environment. Multidrug phenotypes in these populations can therefore provide surveillance signals of cumulative selection and potential co-selection across antimicrobial classes. Formal MDR, XDR, and PDR classification, however, requires an appropriate and sufficiently complete antimicrobial-category framework. Because such a framework could not be applied uniformly to the present retrospective dataset, continuous MIC distributions were used as the primary evidence, the historical multidrug classification was retained only as a sensitivity analysis, and XDR/PDR categories were not assigned [1,2,3].

Commensal *Escherichia coli* is a widely used indicator organism in European food–animal AMR monitoring because standardized susceptibility measurements can capture changes across antimicrobial classes and production systems [3]. Enterococci and staphylococci provide complementary information but differ substantially from *E. coli* in intrinsic susceptibility, ecology and the clinical meaning of particular organism–agent combinations. Consequently, a high minimum inhibitory concentration (MIC) cannot be assigned the same biological interpretation across bacterial groups without an applicable organism-specific clinical breakpoint or epidemiological cutoff value.

Hungarian poultry surveillance has generated both national and regional datasets that make this distinction particularly important. Previous studies of poultry-associated *E. coli* and *Enterococcus* spp. established broad susceptibility baselines [4,5], while farm- and region-resolved investigations have shown that resistance distributions can differ materially across production settings [6,7]. A Hungarian Veterinary Journal analysis of large-scale chicken flocks further demonstrated substantial variation according to production purpose [8]. Regional surveillance therefore has value when it preserves the relationship between isolate identity, production metadata and measured MICs rather than reducing heterogeneous data to a single national percentage.

Genomic work adds a second layer of interpretation. Resistance-associated sequences and mobile genetic elements have been described in *E. coli* from Hungarian layers [9] and in farm animal *E. coli* from other settings [10]. Food-chain studies have likewise documented multidrug-resistant and extended-spectrum beta-lactamase-producing *E. coli* in table eggs [11]. These investigations show why phenotypic and genomic data are complementary, but they also underline a key limitation: co-occurrence of high MICs does not establish physical genetic linkage, and detection of a resistance-associated sequence does not by itself demonstrate expression, transfer, or population prevalence.

Antimicrobial stewardship also requires exposure and management data that cannot be inferred from MIC patterns alone; antimicrobial-use records, biosecurity measures, and environmental surveillance therefore provide complementary information [12,13,14,15,16].

Accordingly, the primary objective of this study was to characterize the regional MIC landscape of chicken-associated *Staphylococcus* spp., *Enterococcus* spp., and *Escherichia coli* from Central Hungary and to determine whether the *E. coli* susceptibility profiles formed structured connected components in a thresholded MIC-correlation network. We hypothesized that recurrent *E. coli* MIC covariation would remain detectable after multiplicity correction and would not be abolished by sensitivity analyses addressing assay-boundary accumulation. The historical multidrug classification was retained only as a secondary sensitivity analysis, whereas continuous MIC measurements were used for the primary statistical inference. Nine publicly deposited Central Hungary *E. coli* assemblies were evaluated separately as a contextual genomic panel. This design was intended to provide a transparent regional analysis relevant to antimicrobial stewardship without converting uncertain retrospective thresholds into unsupported clinical or population-level claims.

## 2. Results

### 2.1. Composition of the Central Hungary Collection

The primary regional phenotype dataset comprised 133 bacterial isolates from adult chickens sampled during routine veterinary visits in 2022–2023. The collection contained 28 *Staphylococcus* spp., 59 *Enterococcus* spp., and 46 *E. coli* isolates. Seventy-three isolates originated from respiratory samples and 60 from cloacal samples. The source composition differed by bacterial group: staphylococci were predominantly respiratory (26/28), enterococci were nearly balanced between respiratory and cloacal sources (28/59 and 31/59), and *E. coli* were more frequently cloacal (27/46) than respiratory (19/46). Production metadata also differed: the 46 *E. coli* isolates in the regional phenotype dataset originated from breeding flocks, whereas the Gram-positive subsets contained both egg-production and breeding-flock isolates (Table 1). These differences preclude an unqualified cross-genus ranking of resistance risk (Supplementary Table S1).

### 2.2. Recorded MIC Distributions and Historical Threshold-Based Sensitivity Analysis

**Figure 1:**
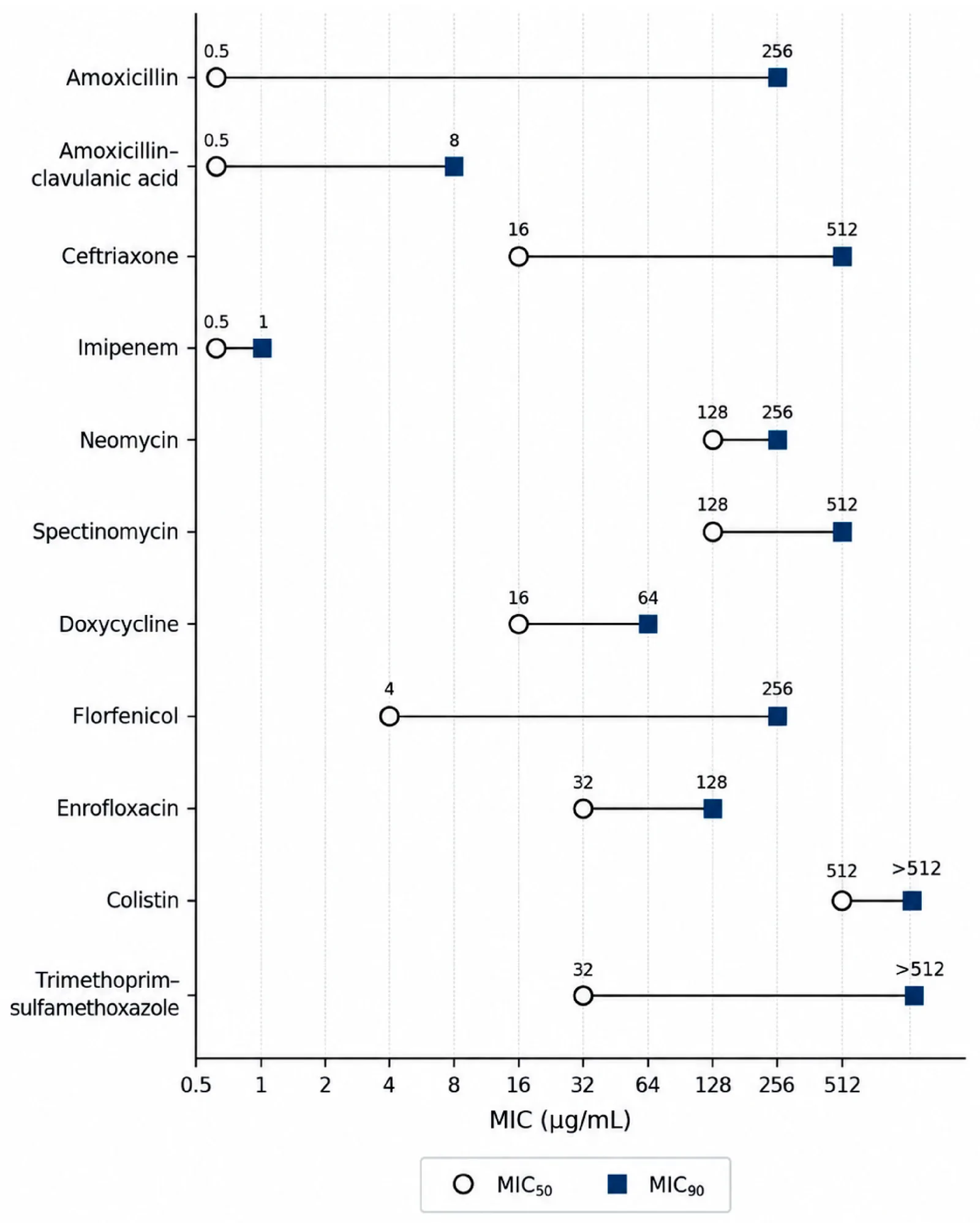
Recorded MIC_50_ and MIC_90_ values for the predefined *Escherichia coli* analytical panel. The x-axis is log2-scaled. Values are shown as observed or right-censored assay endpoints; >512 µg/mL denotes growth at the highest-tested concentration and does not represent an actual measurement at 1024 µg/mL.

MIC distributions were heterogeneous within each bacterial group and across the antimicrobial panel. In *E. coli*, the recorded MIC_50_/MIC_90_ values were 0.5/256 µg/mL for amoxicillin, 16/512 µg/mL for ceftriaxone, 128/256 µg/mL for neomycin, 128/512 µg/mL for spectinomycin, 16/64 µg/mL for doxycycline, 4/256 µg/mL for florfenicol, and 32/128 µg/mL for enrofloxacin. Trimethoprim–sulfamethoxazole showed a recorded MIC_50_/MIC_90_ of 32/>512 µg/mL. The broth-microdilution assay covered a twofold concentration range of 0.001–512 µg/mL. Isolates that continued to grow at the highest-tested concentration of 512 µg/mL had historically been assigned the numerical code 1024 µg/mL in the source workbook. Because 1024 µg/mL was not an actually tested concentration, these observations are reported in the revised manuscript as right-censored MICs of >512 µg/mL. For rank-based analyses, all >512 µg/mL observations were retained as a common ordinal category above 512 µg/mL and remained tied; the numerical code 1024 was not interpreted as an actual measured concentration (Figure 1).

The Gram-positive datasets showed different distributions and included organism–agent combinations for which high MICs are expected because of intrinsic or poorly interpretable susceptibility. For example, colistin values in enterococci and staphylococci and third-generation cephalosporin values in enterococci were retained in the data record but were not interpreted as newly acquired resistance. The full MIC_50_/MIC_90_ matrix is shown in Table 2 (Supplementary Table S4).

The archived regional analysis included a historical binary threshold classification in which 42/46 *E. coli* isolates (91.3%) were threshold-positive in at least three antimicrobial classes. Because the complete provenance and current applicability of the underlying thresholds could not be reconstructed, this value is retained only as a historical sensitivity descriptor and is not interpreted as a validated MDR prevalence estimate. XDR and PDR classifications were not reconstructed [17,18,19].

### 2.3. Continuous MIC Covariation Identifies Two Connected E. coli Network Components

**Figure 2:**
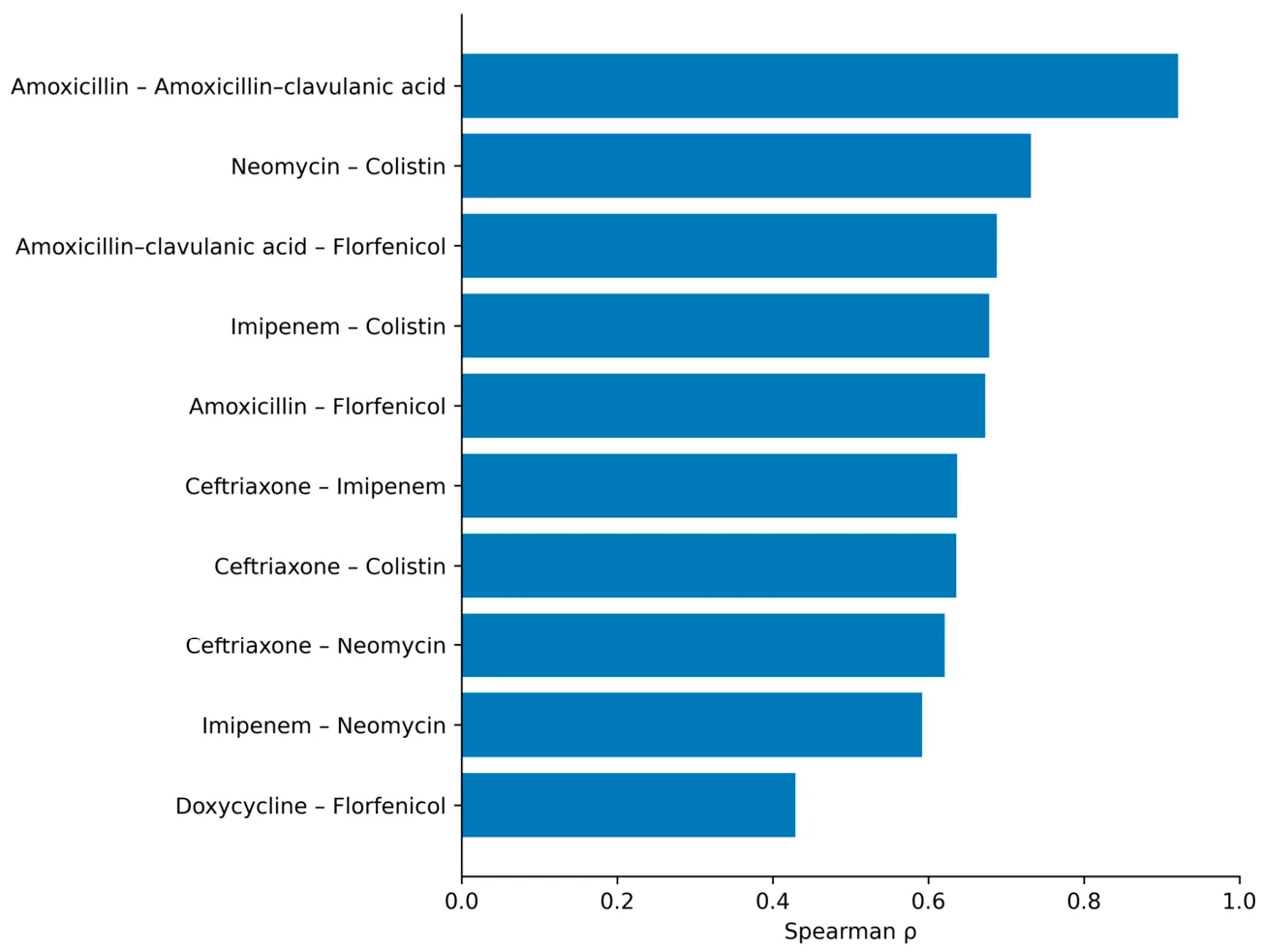
Ranked pairwise Spearman correlations among recorded *Escherichia coli* minimum inhibitory concentrations (MICs) meeting the prespecified |ρ| ≥ 0.40 and Benjamini–Hochberg-adjusted q < 0.05 criteria.

To avoid making the primary network dependent on retrospective binary thresholds, pairwise associations were recalculated directly from isolate-level *E. coli* MIC ranks (Figure 2). After Benjamini–Hochberg false-discovery-rate correction, ten relationships met the prespecified criteria of |ρ| ≥ 0.40 and adjusted q < 0.05 (Table 3; Supplementary Table S2). The strongest association was between amoxicillin and amoxicillin–clavulanic acid (ρ = 0.921), followed by neomycin–colistin (ρ = 0.732), amoxicillin–clavulanic acid–florfenicol (ρ = 0.688), imipenem–colistin (ρ = 0.678), and amoxicillin–florfenicol (ρ = 0.673).

**Figure 3:**
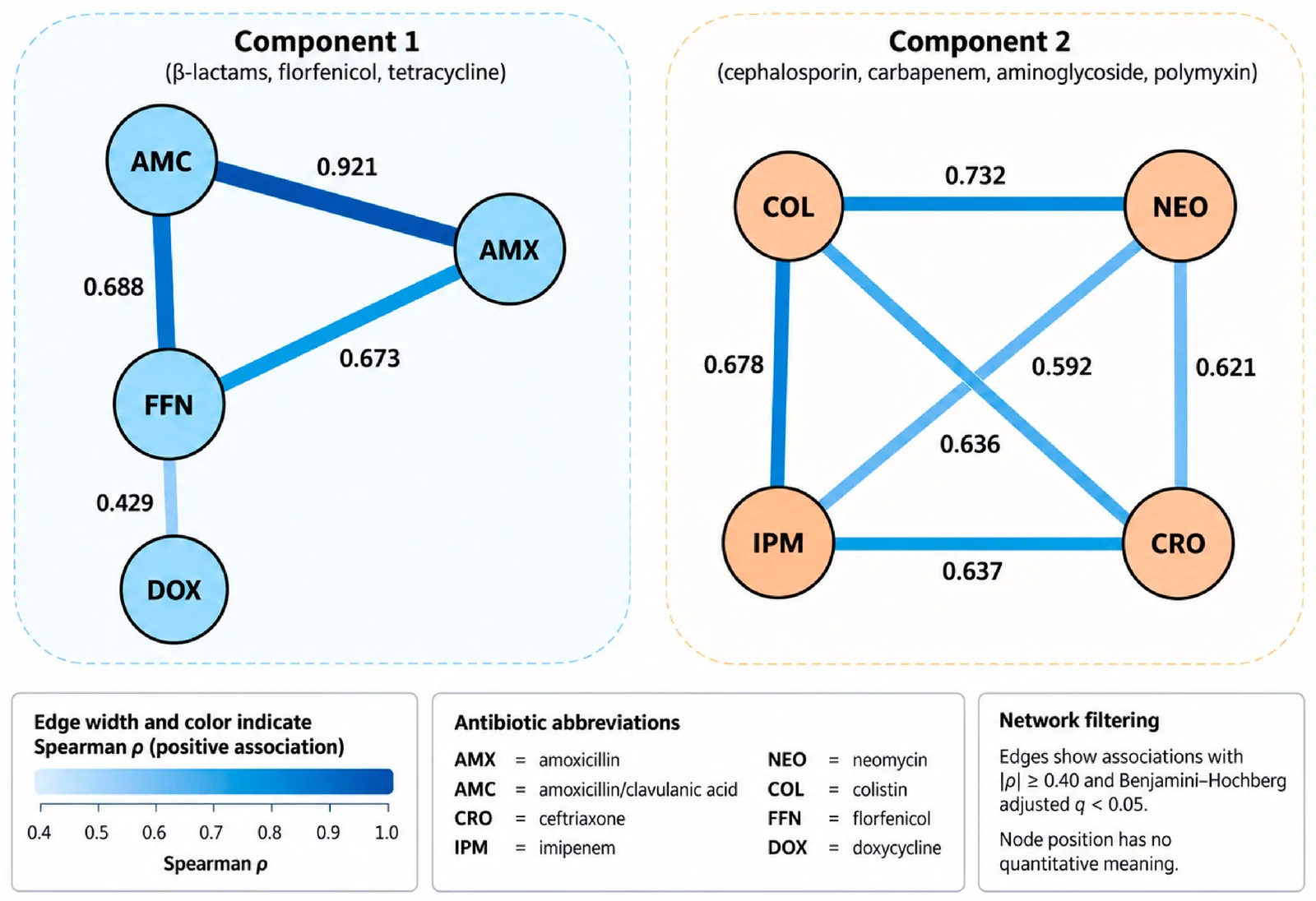
Minimum inhibitory concentration (MIC) covariation network for *Escherichia coli*. Edges represent isolate-level Spearman associations meeting |ρ| ≥ 0.40 and Benjamini–Hochberg-adjusted q < 0.05. Edge width and color intensity were scaled to the correlation coefficient. Node position has no quantitative meaning.

The thresholded network comprised two connected components (Figure 3)—one connected amoxicillin and amoxicillin–clavulanic acid with florfenicol and doxycycline; the other linked ceftriaxone, imipenem, neomycin and colistin. These structures indicate repeated MIC covariation within the sampled isolates but do not establish common plasmid location, shared resistance genes, or causal co-selection. The ceftriaxone–colistin association (ρ = 0.636), for example, represents an isolate-level phenotypic association rather than evidence of physical linkage between β-lactam and polymyxin resistance determinants.

Assessment of assay-boundary accumulation showed no *E. coli* observations at the lower tested limit of 0.001 µg/mL for any of the 11 network agents. Upper-bound accumulation was most pronounced for spectinomycin (13/46, 28.3% at 512 µg/mL), colistin (31/46, 67.4% at or above 512 µg/mL), and trimethoprim–sulfamethoxazole (17/46, 37.0% at or above 512 µg/mL). A sensitivity analysis excluding these three agents retained seven FDR-supported correlations meeting |ρ| ≥ 0.40 and adjusted q < 0.05. Importantly, the two-component network structure persisted: amoxicillin, amoxicillin–clavulanic acid, florfenicol, and doxycycline remained connected, while ceftriaxone, imipenem, and neomycin formed the second component. Thus, the principal network structure was not dependent on the agents showing substantial upper-bound accumulation (Table S5; Figure S1A). The network was also robust to removal of the mechanically related amoxicillin–clavulanic acid variable. After its exclusion, eight associations met the prespecified |ρ| ≥ 0.40 and adjusted q < 0.05 criteria. The first component remained connected through amoxicillin–florfenicol (ρ = 0.673) and doxycycline–florfenicol (ρ = 0.429), whereas the ceftriaxone–imipenem–neomycin–colistin component was unchanged. Thus, the overall two-component structure was not created solely by the strong amoxicillin–amoxicillin/clavulanic acid correlation (Figure S1B).

### 2.4. Contextual Genomic Panel of Nine Central Hungary E. coli Isolates

Nine *E. coli* draft genome assemblies from Central Hungary were available as an independent contextual genomic panel and have been deposited publicly under the National Center for Biotechnology Information (NCBI) BioProject PRJNA1521108. This genomic panel was analyzed separately from the 46-isolate primary regional phenotype denominator and was not assumed to be a statistically representative subsample of that collection. Seven isolates originated from laying flocks and two from breeding flocks; three were respiratory and six cloacal according to the sequencing metadata.

The final repository-prepared assemblies ranged from 4.68 to 5.40 Mb. Assembly continuity was heterogeneous: isolates 763, 766 and 767 had N_50_ values above 100 kb, whereas isolates 757, 761 and 769 remained substantially more fragmented (N_50_ 2.19–3.62 kb). This variation is relevant to negative genomic inference; failure to detect a selected determinant in a fragmented draft genome should not be treated as equivalent to demonstrated gene absence. The genomic panel was retained solely to document whether publicly available Central Hungary *E. coli* genomes displayed a uniform or heterogeneous resistance-gene background and was not intended to explain the 46-isolate MIC network mechanistically. Two genome-panel isolates, 984 and 1015, could be linked unambiguously to records in the primary phenotypic cohort; the remaining seven genomic isolates originated from laying flocks and were outside the 46-isolate breeding-flock phenotype denominator (Table 4; Supplementary Table S3).

**Figure 4:**
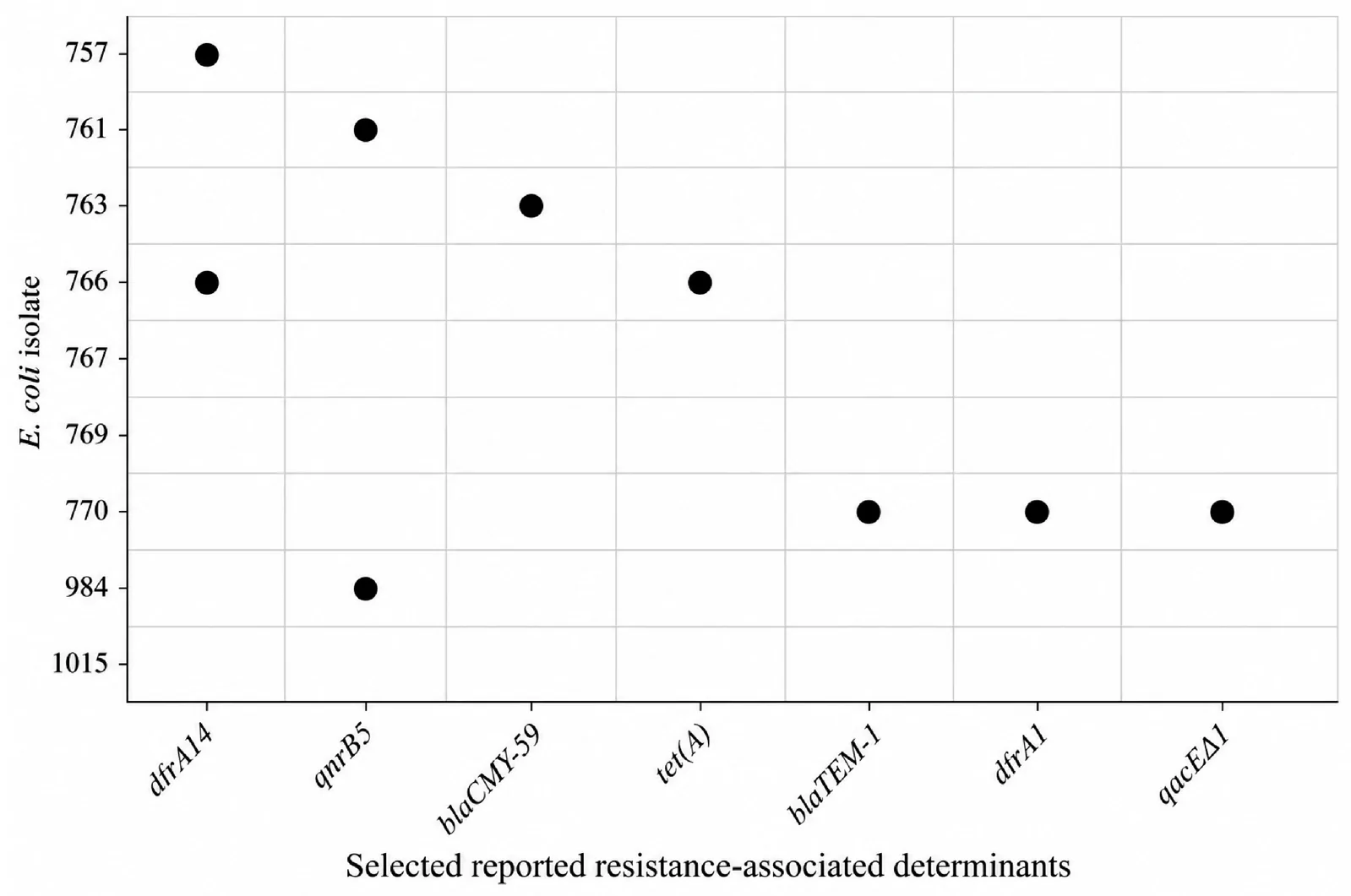
Presence matrix of selected reported resistance-associated determinants in the nine publicly deposited Central Hungary *Escherichia coli* assemblies. Filled cells indicate an archived bioinformatic call; unfilled cells indicate no corresponding selected call in the archived output and should not be interpreted as confirmed gene absence.

The selected reported calls were heterogeneous. *dfrA14* was identified in isolates 757 and 766, *qnrB5* in 761 and 984, *blaCMY-59* in 763, *tet(A)* in 766, and *bla_TEM-1_* together with *dfrA1* and *qacEΔ1* in isolate 770. The remaining three assemblies did not contain one of the selected acquired/resistance-associated calls highlighted in Figure 4. Because the nine genomes constituted a separate contextual panel and were not a representative subset of the 46-isolate phenotypic cohort, they were not used to assign mechanisms to the MIC network. Their role was limited to documenting genomic heterogeneity among publicly available Central Hungary *E. coli* isolates. For the two linked isolates, isolate 984 carried a reported *qnrB5* call and had an enrofloxacin MIC of 64 µg/mL, whereas isolate 1015 had no selected resistance-associated call in the archived panel and an enrofloxacin MIC of 16 µg/mL. Their complete linked MIC profiles are provided in Table S6. Because only two of the nine genomes overlapped with the primary phenotypic cohort, no inferential genotype–phenotype association was performed.

## 3. Discussion

The Central Hungary chicken-associated collection displayed broad and structured *E. coli* MIC variation, with the main finding of the continuous analysis being the organization of MIC covariation into two connected components. A historical threshold analysis also indicated a broad multidrug signal, but because the complete threshold scheme could not be revalidated against a current, uniformly applicable breakpoint/ECOFF framework, the corresponding percentage is not interpreted here as an MDR prevalence estimate.

The *E. coli* MIC distributions were characterized by high upper tails for several compounds. Ceftriaxone, florfenicol, trimethoprim–sulfamethoxazole and colistin all showed wide separation between parts of their recorded distributions, while enrofloxacin had a notably high MIC_50_/MIC_90_ of 32/128 µg/mL in this regional collection. These patterns are compatible with the substantial fluoroquinolone and multidrug resistance reported in poultry *E. coli* elsewhere, but direct percentage comparisons remain sensitive to sampling framework, production type, test panel and interpretive criteria [3,4,6]. The present *E. coli* subset originated from breeding flocks; it should therefore not be treated as interchangeable with slaughterhouse, broiler-caecal or retail-food collections.

The network adds information that a single MDR percentage cannot provide. As expected, amoxicillin and amoxicillin–clavulanic acid were strongly correlated because both measurements contain the same aminopenicillin component; this association should not be interpreted as independent evidence of co-selection. Consistent with this interpretation, exclusion of amoxicillin–clavulanic acid from a sensitivity network did not eliminate the two-component structure. The ceftriaxone–imipenem–neomycin–colistin component should nevertheless be regarded strictly as an exploratory phenotypic covariance pattern and not as evidence of common exposure, shared resistance determinants, therapeutic cross-resistance, or physical genetic linkage. Similar combinations of resistance-associated phenotypes in farm animal *E. coli* have been associated with acquired resistance genes and mobile genetic elements [9,10], yet the current network cannot identify the underlying physical mechanism. Phenotypic covariance can reflect shared genetic elements, clonal background, parallel selection, correlated antimicrobial exposure or combinations of these processes. Some of the observed relationships were expected, whereas others were less intuitive. The strong amoxicillin–amoxicillin/clavulanic acid association was anticipated because both measurements contain the same aminopenicillin component, and its removal did not disrupt the overall network structure. In contrast, the ceftriaxone–imipenem–neomycin–colistin component links agents from distinct antimicrobial classes with different targets and expected resistance mechanisms and was therefore less readily predictable. The high accumulation of colistin, spectinomycin, and trimethoprim–sulfamethoxazole observations at the upper assay boundary was also unexpected and motivated the explicit censoring sensitivity analysis. Persistence of the principal connected components after exclusion of these agents argues against the network being solely an artifact of ceiling effects, although the biological mechanisms underlying the observed covariance remain unresolved.

Studies from neighboring Romania provide useful geographic context, although differences in sampling stage, antimicrobial panels, and interpretive criteria preclude direct numerical comparison. Brătfelan et al. reported high resistance among *E. coli* recovered from Romanian chicken meat, including resistance to tetracycline and ampicillin (80% each), sulfamethoxazole (73.3%), chloramphenicol (70%), nalidixic acid (60%), ciprofloxacin (56.7%), and trimethoprim (50%), whereas colistin resistance was detected in one isolate [20]. More recently, Iancu et al. examined broiler carcass and cecal isolates collected in Romania in 2024 and reported high resistance to tetracycline (82.6%), ampicillin (68.3%), and trimethoprim–sulfamethoxazole (61.2%), together with MDR in 34.3% of isolates and ESBL production in approximately 11% [21]. These findings reinforce the regional relevance of aminopenicillin, tetracycline, sulfonamide, and fluoroquinolone resistance in poultry-associated *E. coli*. However, the Romanian data derived from slaughterhouse and retail or cecal sampling frameworks and should not be interpreted as directly comparable prevalence estimates for the breeding-flock surveillance collection analyzed here.

The nine-genome contextual panel reinforces that caution. Selected resistance-associated calls differed between isolates rather than defining one common resistome signature. The presence of beta-lactamase-, quinolone-, tetracycline- and folate-pathway-associated calls in different combinations is consistent with multiple genomic routes to resistance-associated phenotypes. At the same time, assembly continuity varied markedly. Positive sequence evidence can be informative even in a fragmented assembly, but negative calls become progressively less secure as continuity deteriorates. This distinction is especially important when interpreting draft assemblies in surveillance studies and is consistent with broader genomic work on farm animal *E. coli* [9,10,11].

The Gram-positive isolates provide useful regional context but require a different interpretive frame. Enterococci and staphylococci have intrinsic susceptibility characteristics that make some E. coli-oriented agent comparisons biologically inappropriate. Studies of poultry enterococci and staphylococci similarly emphasize species composition and production context [22,23,24]. For this reason, we report their MIC distributions but do not use high values for intrinsically unsuitable combinations to inflate a cross-genus resistance ranking. For the same reason, XDR and PDR categories were not assigned because the tested panel did not provide a complete, uniformly applicable framework for those classifications.

### 3.1. Scientific Contribution and Practical Implications

From a stewardship perspective, the network should be viewed as a prioritization tool rather than a treatment algorithm. The strong associations identify drug pairs and connected components for which common exposure histories, co-selection or shared determinants deserve targeted investigation. Demonstrating those drivers would require antimicrobial-use data and a sampling design that supports farm-level or longitudinal analysis. The substantial reductions in antimicrobial use recently documented on Hungarian pig farms show what direct consumption monitoring can contribute, but those observations cannot be substituted for poultry exposure data [12].

The present study contributes three elements to regional poultry AMR surveillance. First, it demonstrates that continuous MIC data can reveal structured antimicrobial covariation without requiring every organism–agent combination to be forced into a binary susceptible/resistant category. Second, the principal network structure remained detectable after sensitivity analyses addressing both assay-boundary accumulation and the mechanically related amoxicillin–amoxicillin/clavulanic acid measurements, providing greater confidence that the observed pattern was not generated by a single analytical artifact. Third, the study separates phenotypic covariance from genomic inference by treating the nine-genome dataset strictly as contextual information. From a practical perspective, the network is not a treatment algorithm; rather, it identifies antimicrobial pairs and classes that warrant targeted investigation using contemporaneous antimicrobial-use data, longitudinal sampling, and matched genomic analysis.

Prevention remains equally important. Regional AMR surveillance can inform biosecurity priorities, but it does not test specific interventions. Stabilized hypochlorous acid has been reviewed as a modern hygiene option [13], and antimicrobial peptides are being investigated as anti-infective and gut-health tools, including in poultry [14,15]. Likewise, wild birds may participate in AMR dissemination networks at the livestock–environment interface [16]. None of these mechanisms or interventions was evaluated in the present study; they are relevant as future directions rather than explanations of the observed Central Hungary pattern.

### 3.2. Limitations and Future Directions

Several limitations define the boundaries of inference. First, the analytical unit was the bacterial isolate, whereas complete farm-level dependence structures were not available for hierarchical modeling. The reported percentages therefore describe the sampled collection, not a probability-based regional prevalence. Second, a complete, versioned interpretive scheme could not be reconstructed for every organism–agent combination, so continuous MIC distributions were prioritized and the archived multidrug classification was retained only as a sensitivity analysis. Third, the genomic panel of nine isolates was analyzed separately and was not assumed to represent the 46-isolate phenotype cohort. Fourth, genomic analysis was based on draft assemblies; it was not designed to resolve complete plasmids or prove transfer. These restrictions are deliberate and prevent the stronger claims that the available evidence cannot support.

Future studies should therefore use farm-stratified or longitudinal sampling designs that preserve within-farm dependence, collect contemporaneous antimicrobial-use and management data, and employ predefined dilution ranges that minimize endpoint censoring. Genomic analyses would be most informative if performed on the same isolates used for MIC-network construction, with larger matched phenotype–genotype datasets and, where mechanistic linkage is a primary objective, long-read sequencing to resolve plasmid and chromosomal context. Replication in independent poultry populations will also be required to determine whether the connected MIC components identified here are stable regional features or sample-specific structures.

Within those boundaries, the study provides a coherent regional signal. The high historical threshold-positive classification in *E. coli*, broad MIC distributions, and FDR-supported network components all point in the same direction: the sampled Central Hungary *E. coli* collection contained an extensive multidrug phenotypic structure. The nine-genome panel illustrates genomic heterogeneity but cannot establish the genetic basis of the MIC correlations observed in the separate phenotypic cohort. Their role was limited to documenting genomic heterogeneity among publicly available Central Hungary *E. coli* isolates.

## 4. Materials and Methods

### 4.1. Study Design, Sampling and Analytical Denominators

**Figure 5:**
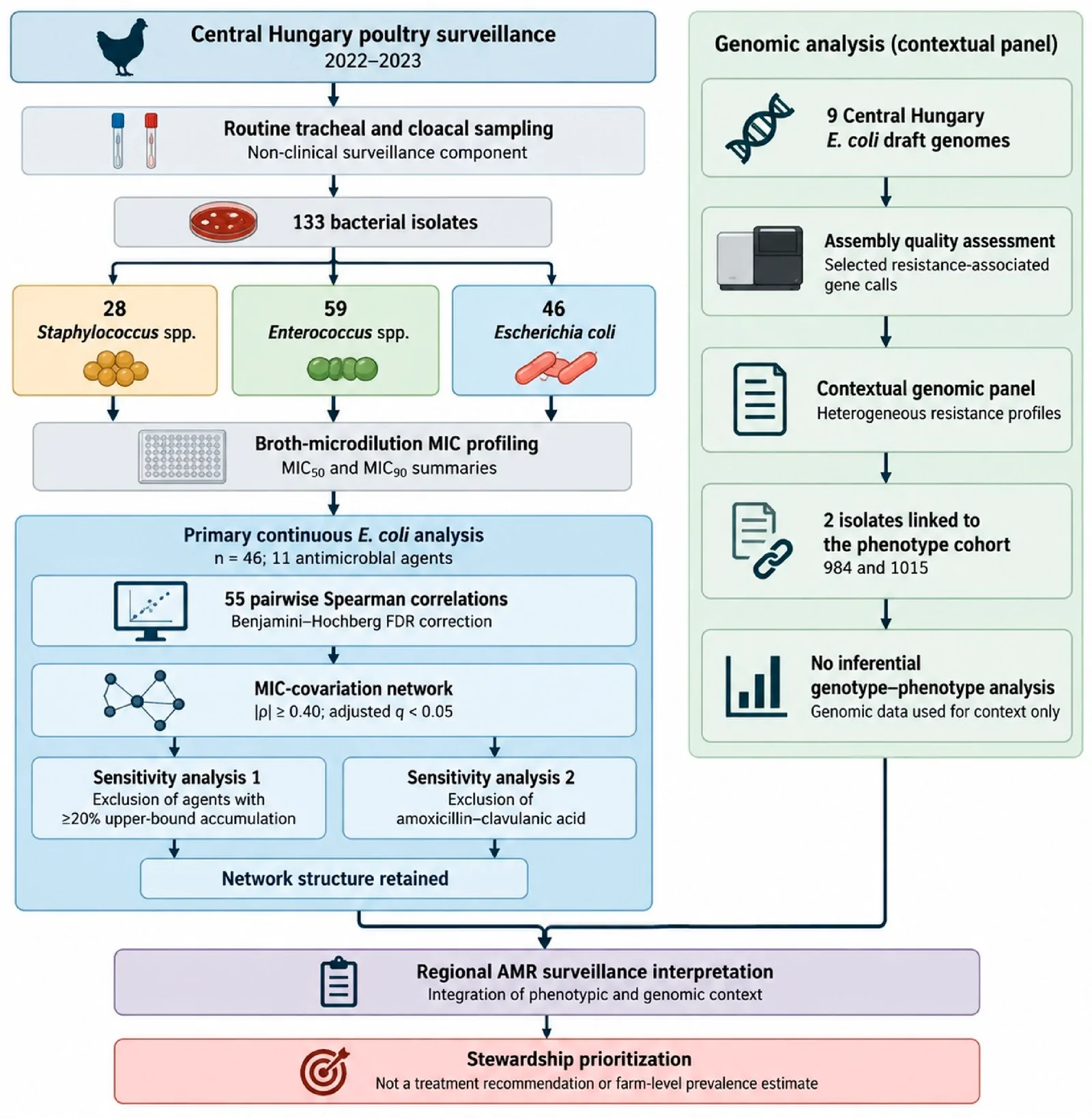
Overview of the study design and analytical workflow. The primary regional phenotype dataset comprised 133 chicken-associated bacterial isolates, with continuous MIC-network analysis restricted to the 46 *Escherichia coli* isolates. The nine-genome dataset was analyzed separately as a contextual genomic panel; only two genomic isolates could be linked to the primary phenotypic cohort, and no inferential genotype–phenotype analysis was performed. FDR, false discovery rate; MIC, minimum inhibitory concentration.

This observational laboratory study investigated chicken-associated bacterial isolates obtained from large-scale chicken flocks in Central Hungary during 2022–2023. The broader sampling program comprised a non-clinical surveillance arm and a separate clinical-case collection. According to the source study records, tracheal and cloacal samples constituted the commensal-surveillance component, whereas isolates from clinical cases were collected separately from pathological sites and were not eligible for inclusion in the present regional analysis. The present cohort therefore contained no isolates obtained from lesions or from cases selected because of a culture-confirmed infection caused by the target organism. The term “commensal” is used only when referring to the original surveillance classification and does not imply that every recovered organism was permanently non-pathogenic to its host. The overall study design and analytical workflow are summarized in Figure 5.

The primary regional phenotype dataset was defined by the exact host and region fields in the source MIC workbook and comprised 133 isolates: 28 *Staphylococcus* spp., 59 *Enterococcus* spp., and 46 *E. coli*. No farm-level or population-prevalence inference was made from these isolate counts. A separate genomic context panel consisted of nine Central Hungary *E. coli* isolates for which draft assemblies and sequencing metadata were available. The genomic panel was not treated as a representative random subset of the 46-isolate regional *E. coli* phenotype denominator.

The Animal Welfare Committee of the University of Veterinary Medicine Budapest issued a certificate of exemption on 19 May 2025 for the project “Antimicrobial Resistance in Commensal Bacteria from Large-Scale Chicken Flocks in the Közép–Magyarország Region of Hungary”. The committee confirmed that routine diagnostic tracheal and cloacal swab collection did not constitute an animal experiment under Act XXVIII of 1998 and Government Decree No. 40 of 2013. No experimental infection, research-directed treatment allocation or additional invasive animal manipulation was performed.

### 4.2. Bacterial Isolation, Identification and Antimicrobial Compounds

Samples were processed at the Microbiology Laboratory of the Department of Pharmacology and Toxicology, University of Veterinary Medicine Budapest. *Staphylococcus* spp. were isolated on CHROMagar Staph aureus agar (Chebio Fejlesztő Kft., Budapest, Hungary), *E. coli* on ChromoBio Coliform agar (Biolab Zrt., Budapest, Hungary), and enterococci on modified m-*Enterococcus* agar (Merck KGaA, Darmstadt, Germany). Presumptive colonies were subcultured on tryptic soy agar to obtain pure cultures and stored in a Microbank system (Pro-Lab Diagnostics, Richmond Hill, ON, Canada) at −80 °C. Species identification was performed by MALDI-TOF mass spectrometry using a Bruker MALDI Biotyper system and Biotyper software version 12.0 (Bruker Daltonics GmbH, Bremen, Germany), applying the manufacturer’s recommended score thresholds.

Analytical-grade antimicrobial reference powders were obtained from Merck KGaA. Stock solutions were prepared in accordance with CLSI dilution-testing recommendations [19]. The panel comprised amoxicillin, amoxicillin–clavulanic acid, ceftriaxone, imipenem, neomycin, spectinomycin, doxycycline, florfenicol, tylosin, tiamulin, lincomycin, enrofloxacin, colistin, trimethoprim–sulfamethoxazole and vancomycin. The antimicrobial panel was designed as a broad surveillance panel rather than as a therapeutic recommendation panel. It included agents representing antimicrobial classes relevant to poultry production and veterinary AMR monitoring, together with sentinel compounds from clinically important classes that allowed cross-class MIC patterns to be examined. The panel therefore covered aminopenicillins and β-lactamase-inhibitor combinations, cephalosporins, carbapenems, aminoglycosides/aminocyclitols, tetracyclines, phenicols, macrolides, pleuromutilins, lincosamides, fluoroquinolones, polymyxins, potentiated sulfonamides, and glycopeptides. Inclusion of an agent in the surveillance panel does not imply authorization or routine therapeutic use in poultry [4,5,8]. Amoxicillin and amoxicillin–clavulanic acid (2:1) were prepared in 0.01 mol/L phosphate buffer at pH 7.2; imipenem was prepared in 0.1 mol/L phosphate buffer at pH 6.0. Ceftriaxone, doxycycline, spectinomycin, neomycin, colistin, tiamulin, tylosin, lincomycin and vancomycin were dissolved in distilled water. Trimethoprim and sulfamethoxazole were prepared at a 1:19 ratio using the acid/base solvents specified in the laboratory protocol, enrofloxacin was dissolved with a small amount of 1 mol/L NaOH, and florfenicol with a small volume of 95% ethanol followed by distilled water. Stocks were prepared at 1024 µg/mL after potency correction.

### 4.3. Broth-Microdilution MIC Testing and Interpretive Strategy

MICs were determined by broth microdilution according to CLSI methodology for aerobic bacteria [19]. Frozen isolates were recovered in cation-adjusted Mueller–Hinton broth and incubated at 37 °C for 18–24 h. Twofold antimicrobial dilution series covering 0.001–512 µg/mL were prepared in sterile 96-well microtiter plates. The same analytical concentration range was used for all 15 antimicrobial agents. Growth persisting at 512 µg/mL was historically encoded as 1024 µg/mL in the analytical workbook; throughout the revised manuscript, these entries are treated as right-censored values of >512 µg/mL rather than as measurements performed at 1024 µg/mL. Column 11 served as the positive growth control and Column 12 as the sterility control. Bacterial suspensions were adjusted to 0.5 McFarland and diluted 1:20, and inoculated to yield approximately 5 × 10^5^ CFU/mL. Plates were incubated aerobically at 37 °C for 18–24 h. MIC endpoints were the lowest concentrations completely inhibiting visible growth and were read using an SWIN automatic MIC reader and VIZION system (CheBio Fejlesztő Kft., Budapest, Hungary). Quality control was performed on each testing day using *Escherichia coli* ATCC 25922, *Enterococcus faecalis* ATCC 29212, and *Staphylococcus aureus* ATCC 29213; in all testing runs, the observed MICs of the quality-control strains fell within the applicable CLSI-recommended acceptance ranges for the antimicrobial agents tested.

Where applicable, CLSI clinical breakpoints and EUCAST interpretive criteria or epidemiological cutoff concepts were used as surveillance context [17,19]. However, because a complete and internally consistent applicable breakpoint/ECOFF set could not be established retrospectively for all organism–antimicrobial combinations, the primary analysis did not dichotomize all MICs into universal susceptible/resistant categories. MIC_50_ and MIC_90_ were calculated as observed order statistics. Organism–agent combinations with intrinsic or biologically inappropriate susceptibility interpretation were retained in the raw record but were not used to infer newly acquired resistance.

The archived regional threshold analysis was retained as a sensitivity analysis. In that historical output, multidrug resistance was operationally defined as threshold positivity to at least one agent in three or more antimicrobial classes, consistent in structure with the international MDR terminology [18]. Because the provenance and applicability of every individual historic threshold could not be re-established to current standards, the resulting MDR percentage is explicitly described as operational rather than a validated clinical or population-prevalence estimate. XDR and PDR categories were not assigned in the present reanalysis.

### 4.4. Continuous MIC Correlation and Network Analysis

The primary *E. coli* network analysis was conducted on recorded continuous MICs rather than the archived binary classifications. The predefined analytical panel comprised amoxicillin, amoxicillin–clavulanic acid, ceftriaxone, imipenem, neomycin, spectinomycin, doxycycline, florfenicol, enrofloxacin, colistin and trimethoprim–sulfamethoxazole. The network panel was restricted to agents with biologically interpretable activity against *E. coli* and informative variation in the regional dataset; tylosin, tiamulin, lincomycin and vancomycin were excluded because their MIC distributions were not used for acquired resistance inference in *E. coli*. Pairwise Spearman rank correlations were calculated across the 46 *E. coli* isolates. Two-sided *p* values were adjusted across the pairwise comparisons using the Benjamini–Hochberg procedure [25]. All 55 pairwise comparisons among the 11 antimicrobial agents were included in the Benjamini–Hochberg adjustment. Because twofold MIC series produce tied ranks and may include values at assay boundaries, potential floor and ceiling effects were evaluated explicitly. For each antimicrobial, we quantified the number and proportion of *E. coli* observations at the lower tested concentration (0.001 µg/mL), at the upper tested concentration (512 µg/mL), and above the upper assay limit (>512 µg/mL). Spearman coefficients used average ranks for tied observations, and all >512 µg/mL values were treated as a common right-censored rank above 512 µg/mL. As a reviewer-motivated sensitivity analysis, agents for which ≥20% of observations occurred at or above the upper assay boundary were excluded, after which all pairwise correlations and Benjamini–Hochberg adjustments were recalculated. This criterion excluded spectinomycin, colistin, and trimethoprim–sulfamethoxazole, leaving eight agents and 28 pairwise comparisons. Statistical analyses were performed in Python 3.13.5 using SciPy 1.17.0 and statsmodels 0.14.6; network construction and visualization used NetworkX 3.6.1 and Matplotlib 3.10.8. Right-censored values above the upper assay limit were retained as a common ordinal category (>512 µg/mL) for rank-based analysis and were not interpreted as exact concentrations. For the graphical network, edges were retained when |ρ| ≥ 0.40 and the adjusted q value was <0.05. Edge width was scaled to the correlation coefficient. The network was interpreted as a phenotypic covariance structure, not as evidence of genetic linkage, common plasmid carriage or causal co-selection.

A second sensitivity analysis addressed the mechanically related amoxicillin and amoxicillin–clavulanic acid measurements. Because both assays contain the same aminopenicillin component, the network analysis was repeated after complete exclusion of the amoxicillin–clavulanic acid variable, with Benjamini–Hochberg adjustment recalculated across the remaining 45 pairwise comparisons.

### 4.5. Whole-Genome Sequencing and Contextual Genomic Analysis

Genomic DNA was extracted from selected *E. coli* cultures using the Quick-DNA Fungal/Bacterial Miniprep Kit (Zymo Research, Irvine, CA, USA), with mechanical disruption using a TissueLyzer LT (Qiagen GmbH, Hilden, Germany). Libraries were prepared from 50 ng DNA using the TruePrep DNA Library Prep Kit V2 for Illumina (Vazyme Biotech, Nanjing, China), indexed, converted to MGI-compatible libraries using the MGIEasy Universal Library Conversion Kit, and sequenced on a DNBSEQ-G400 platform (MGI Tech, Wuhan, China) with 150 bp paired-end chemistry.

Raw read quality was assessed using FastQC v0.11.9 [26]. Reads were processed with fastp v0.23.2-3 [27], Bloocoo v1.0.7 [28] and Trim Galore v0.6.6 [29]. De novo assembly was performed independently with MEGAHIT v1.2.9 [30] and SPAdes v4.0.0 [31], followed by assembly merging with GAM-NGS v1.1b [32]. Assembly quality was assessed with QUAST v5.2 [33] and BUSCO v5 [34], with genome characteristics additionally examined using GenomeScope v2.2 [35]. For repository preparation, FASTA files were standardized and contigs shorter than 200 bp were removed; no assembly was truncated to force an expected *E. coli* genome size. The nine assemblies used in the contextual panel are publicly available through NCBI BioProject PRJNA1521108.

Open reading frames were identified using Prodigal v2.6.3 [36]. Resistance-associated determinants were screened in the archived workflow using Resistance Gene Identifier against CARD and ABRicate with CARD as the nucleotide reference [37,38]. The present manuscript reports selected acquired or otherwise epidemiologically relevant calls while avoiding the automatic interpretation of conserved efflux components and global regulators as acquired resistance determinants. Gene non-detection was not considered definitive evidence of absence in fragmented draft assemblies.

### 4.6. Statistical Reporting and Reproducibility

Analyses were performed at isolate level. No statistical model was used to estimate farm-level or regional population prevalence because the sampling structure did not support independent farm-level inference. MIC summaries and network statistics were recalculated from the isolate-level regional data, and all denominators are stated explicitly. The genomic contextual panel was analyzed with a separate denominator of nine. Reported correlations, q values, assembly metrics and gene-call summaries were checked against their respective source tables, and all figures were generated from the final verified analysis outputs.

## 5. Conclusions

Chicken-associated *E. coli* sampled in Central Hungary displayed broad MIC variation with a structured pattern of covariation. The primary continuous analysis identified two FDR-supported connected components in the MIC-correlation network, led by amoxicillin–florfenicol-related associations and ceftriaxone–imipenem–neomycin–colistin relationships. These networks describe the sampled phenotype structure without implying physical linkage of resistance determinants. A separate nine-genome contextual panel demonstrated heterogeneous resistance-associated gene calls and variable assembly continuity and is publicly available under PRJNA1521108. Together, the findings show that continuous MIC-network analysis can add structure to regional AMR surveillance by identifying antimicrobial relationships that merit targeted follow-up. These relationships should not guide empirical treatment directly; their practical value is to prioritize combinations for subsequent studies integrating antimicrobial-use records, longitudinal sampling, and matched genomic analysis. The results should not be interpreted as a probability-based prevalence estimate for all Central Hungary chicken farms or as an empirical treatment recommendation.

## Figures and Tables

**Table 1:** Composition of the Central Hungary chicken-associated regional isolate collection.

Bacterial Group	Isolates	Respiratory	Cloacal	Egg Production	Breeding
*Staphylococcus* spp.	28	26	2	15	13
*Enterococcus* spp.	59	28	31	26	33
*Escherichia coli*	46	19	27	0	46
Total	133	73	60	41	92

All isolates were recorded as originating from adult chickens. Isolate counts are analytical isolate denominators, not independent farm or animal denominators. No isolates obtained from pathological lesions or from the separately collected clinical-case dataset were included in the present regional cohort.

**Table 2:** Recorded MIC_50_/MIC_90_ values (µg/mL) in the Central Hungary collection.

Antimicrobial	*Staphylococcus* spp. (n = 28)	*Enterococcus* spp. (n = 59)	*Escherichia coli* (n = 46)
Amoxicillin	0.25/1	1/256	0.5/256
Amoxicillin–clavulanic acid	0.125/2	1/8	0.5/8
Ceftriaxone	1/>512	64/>512	16/512
Imipenem	0.125/4	0.5/4	0.5/1
Neomycin	32/256	128/>512	128/256
Spectinomycin	32/256	128/512	128/512
Doxycycline	1/64	16/128	16/64
Florfenicol	4/8	8/64	4/256
Tylosin	1/>512	512/>512	512/>512
Tiamulin	32/512	256/512	256/512
Lincomycin	16/>512	512/>512	512/>512
Enrofloxacin	2/32	1/32	32/128
Colistin	512/>512	>512/>512	512/>512
Trimethoprim–sulfamethoxazole	4/256	4/>512	32/>512
Vancomycin	0.25/64	4/512	1/256

MIC_50_ and MIC_90_ are observed order statistics. Values are presented as observed or right-censored assay endpoints; >512 µg/mL denotes growth at the highest-tested concentration and does not represent an actual measurement at 1024 µg/mL. These values do not by themselves constitute universal clinical susceptibility categories. Intrinsically unsuitable organism–agent combinations are retained for traceability but are not interpreted as evidence of acquired resistance.

**Table 3:** Pairwise *Escherichia coli* minimum inhibitory concentration (MIC) correlations meeting the prespecified |ρ| ≥ 0.40 and Benjamini–Hochberg-adjusted q < 0.05 criteria.

Antimicrobial 1	Antimicrobial 2	Spearman ρ	BH-Adjusted q
Amoxicillin	Amoxicillin–clavulanic acid	0.921	6.77 × 10^−18^
Neomycin	Colistin	0.732	2.10 × 10^−7^
Amoxicillin–clavulanic acid	Florfenicol	0.688	2.26 × 10^−6^
Imipenem	Colistin	0.678	3.16 × 10^−6^
Amoxicillin	Florfenicol	0.673	3.26 × 10^−6^
Ceftriaxone	Imipenem	0.637	1.63 × 10^−5^
Ceftriaxone	Colistin	0.636	1.63 × 10^−5^
Ceftriaxone	Neomycin	0.621	2.88 × 10^−5^
Imipenem	Neomycin	0.592	8.89 × 10^−5^
Doxycycline	Florfenicol	0.429	0.0160

Because within-farm dependence could not be modeled, ρ and q values are exploratory isolate-level statistics and should not be interpreted as farm-adjusted population-level inference.

**Table 4:** Metadata, assembly continuity and selected resistance-associated calls in the nine-genome contextual panel.

Isolate	Source	Production	Assembly (Mb)	Contigs	N_50_ (bp)	Selected Reported Call(s)
757	Respiratory	Laying	4.830	3138	3210	*dfrA14*
761	Respiratory	Laying	4.682	2541	3620	*qnrB5*
763	Respiratory	Laying	5.008	236	103,011	*blaCMY-59*
766	Cloacal	Laying	5.143	280	160,152	*dfrA14*; *tet(A)*
767	Cloacal	Laying	4.887	204	106,360	No selected call
769	Cloacal	Laying	4.770	4100	2189	No selected call
770	Cloacal	Laying	5.157	2421	5468	*bla_TEM-1_*; *dfrA1*; *qacEΔ1*
984	Cloacal	Breeding	5.398	1683	13,890	*qnrB5*
1015	Cloacal	Breeding	5.347	1214	13,459	No selected call

Assemblies were prepared for repository submission by removing contigs shorter than 200 bp and standardizing FASTA formatting. “No selected call” means that none of the determinants highlighted in this table was present in the archived output; it is not proof of genome-wide absence. Gene calls are reported as assembly-linked bioinformatic evidence.

## Data Availability

The nine *Escherichia coli* draft genome assemblies generated for the Central Hungary genomic component are publicly available in the National Center for Biotechnology Information under BioProject accession PRJNA1521108. Aggregated phenotypic results and analysis summaries are provided in the Supplementary Materials. The de-identified isolate-level MIC dataset and analysis code are available from the corresponding author upon reasonable request.

## Supplementary Materials

The following supporting information can be downloaded at https://www.mdpi.com/article/10.3390/antibiotics15100999/s1, Table S1: Complete regional cohort summary; Table S2: *Escherichia coli* correlations retained in the final minimum inhibitory concentration (MIC) network.; Table S3: Nine-genome contextual panel; Table S4: Full recorded MIC_50_/MIC_90_ matrix; Table S5: Assay-boundary distribution of the 46 Central Hungary *Escherichia coli* MIC profiles; Table S6: Isolate-level MIC profiles of the two genomic-panel isolates linked to the primary Central Hungary phenotypic cohort; Figure S1. Sensitivity analyses of the *Escherichia coli* MIC-covariation network. (A) Network after exclusion of agents with ≥20% of observations at or above the upper assay boundary (spectinomycin, colistin, and trimethoprim–sulfamethoxazole). (B) Network after exclusion of the amoxicillin–clavulanic acid variable. In both analyses, edges indicate |ρ| ≥ 0.40 and Benjamini–Hochberg-adjusted q < 0.05.

## Abbreviations

The following abbreviations are used in this manuscript:

AMR	Antimicrobial resistance
CARD	Comprehensive Antibiotic Resistance Database
CLSI	Clinical and Laboratory Standards Institute
ECOFF	Epidemiological cutoff value
FDR	False discovery rate
MDR	Multidrug resistance
MIC	Minimum inhibitory concentration
N50	Contig length at which half of the assembly is contained in contigs of that length or longer
WGS	Whole-genome sequencing
