# Supplementary material for: Network Analysis of Antimicrobial MIC Profiles Reveals Extensive Covariation in Chicken-Associated Escherichia coli from Large-Scale Breeding Flocks in Central Hungary
Source: Antibiotics (Basel). 2026 Oct 9;15(10):999. doi: 10.3390/antibiotics15100999 (PMC13700753; doi:10.3390/antibiotics15100999)
Supplement: Supplementary file 1 [file antibiotics-15-00999-s001.zip › antibiotics-4603471-supplementary.pdf]

Supplementary Material

Supplementary Table S1. Complete regional cohort summary.

Group	n	Respiratory	Cloacal	Egg production	Breeding
Staphylococcus spp.	28	26	2	15	13
Enterococcus spp.	59	28	31	26	33
Escherichia coli	46	19	27	0	46
Total	133	73	60	41	92

All isolates originated from adult chickens.

Supplementary Table S2. *Escherichia coli* correlations retained in the final minimum inhibitory concentration (MIC) network.

Agent 1	Agent 2	Spearman ϱ	BH q
Amoxicillin	Amoxicillin–clavulanic acid	0.921	6.77×10^{-18}
Neomycin	Colistin	0.732	2.10×10^{-7}
Amoxicillin–clavulanic acid	Florfenicol	0.688	2.26×10^{-6}
Imipenem	Colistin	0.678	3.16×10^{-6}
Amoxicillin	Florfenicol	0.673	3.26×10^{-6}
Ceftriaxone	Imipenem	0.637	1.63×10^{-5}
Ceftriaxone	Colistin	0.636	1.63×10^{-5}
Ceftriaxone	Neomycin	0.621	2.88×10^{-5}
Imipenem	Neomycin	0.592	8.89×10^{-5}
Doxycycline	Florfenicol	0.429	0.0160

Selection criterion: $|\varrho| \geq 0.40$ and BH-adjusted $q<0.05$.

Supplementary Table S3. Nine-genome contextual panel.

Isolate	Source	Production	Assembly (Mb)	Contigs	N50 (bp)	Selected reported call(s)
757	Respiratory	Laying	4.830	3,138	3,210	*dfrA14*
761	Respiratory	Laying	4.682	2,541	3,620	*qnrB5*
763	Respiratory	Laying	5.008	236	103,011	*blaCMY-59*
766	Cloacal	Laying	5.143	280	160,152	*dfrA14*; *tet(A)*
767	Cloacal	Laying	4.887	204	106,360	No selected call
769	Cloacal	Laying	4.770	4,100	2,189	No selected call
770	Cloacal	Laying	5.157	2,421	5,468	*blaTEM-1*; *dfrA1*; *qacEΔ1*
984	Cloacal	Breeding	5.398	1,683	13,890	*qnrB5*
1015	Cloacal	Breeding	5.347	1,214	13,459	No selected call

The genomic panel is separate from the 46-isolate primary phenotypic denominator. “No selected call” does not establish gene absence.

Supplementary Table S4. Full recorded MIC_{50}/MIC_{90} matrix (μg/mL).

Antimicrobial	*Staphylococcus spp.*	*Enterococcus spp.*	*Escherichia coli*
Amoxicillin	0.25 / 1	1 / 256	0.5 / 256
Amoxicillin–clavulanic acid	0.125 / 2	1 / 8	0.5 / 8
Ceftriaxone	1 / >512	64 / >512	16 / 512
Imipenem	0.125 / 4	0.5 / 4	0.5 / 1
Neomycin	32 / 256	128 / >512	128 / 256
Spectinomycin	32 / 256	128 / 512	128 / 512
Doxycycline	1 / 64	16 / 128	16 / 64
Florfenicol	4 / 8	8 / 64	4 / 256
Tylosin	1 / >512	512 / >512	512 / >512
Tiamulin	32 / 512	256 / 512	256 / 512
Lincomycin	16 / >512	512 / >512	512 / >512
Enrofloxacin	2 / 32	1 / 32	32 / 128
Colistin	512 / >512	>512 / >512	512 / >512
Trimethoprim–sulfamethoxazole	4 / 256	4 / >512	32 / >512
Vancomycin	0.25 / 64	4 / 512	1 / 256

Values are observed order statistics. Intrinsically unsuitable organism–agent combinations are retained for traceability but are not interpreted as acquired resistance.

Supplementary Table S5. Assay-boundary distribution of the 46 Central Hungary *Escherichia coli* MIC profiles.

Antimicrobial	Tested range	At 0.001	At 512	>512	At/above upper boundary
Amoxicillin	0.001–512	0/46	1/46 (2.2%)	0	1/46 (2.2%)
Amoxicillin–clavulanic acid	0.001–512	0	0	0	0
Ceftriaxone	0.001–512	0	6/46 (13.0%)	3/46 (6.5%)	9/46 (19.6%)
Imipenem	0.001–512	0	0	0	0
Neomycin	0.001–512	0	3/46 (6.5%)	0	3/46 (6.5%)
Spectinomycin	0.001–512	0	13/46 (28.3%)	0	13/46 (28.3%)
Doxycycline	0.001–512	0	0	0	0
Florfenicol	0.001–512	0	0	0	0
Enrofloxacin	0.001–512	0	1/46 (2.2%)	0	1/46 (2.2%)
Colistin	0.001–512	0	15/46 (32.6%)	16/46 (34.8%)	31/46 (67.4%)
Trimethoprim–sulfamethoxazole	0.001–512	0	2/46 (4.3%)	15/46 (32.6%)	17/46 (37.0%)

Values reported as >512 μg/mL were historically encoded as 1024 μg/mL in the source workbook but represent right-censored observations above the highest tested concentration. The sensitivity-analysis exclusion criterion was ≥20% of observations at or above the upper assay boundary.

Supplementary Table S6. Isolate-level MIC profiles of the two genomic-panel isolates that could be linked to the primary Central Hungary phenotypic cohort.

Isolate	AMX	AMC	CRO	IPM	NEO	SPT	DOX	FFN	ENR	COL	SXT	Selected call
**984**	0.125	0.125	128	0.5	128	128	16	4	64	512	2	*qnrB5*
**1015**	256	8	0.063	0.125	128	128	128	256	16	0.063	>512	No selected call

MICs are expressed in μg/mL. >512 indicates growth at the highest tested concentration and is a right-censored endpoint. “No selected call” denotes absence of the determinants highlighted in the archived selected-gene panel and is not evidence of genome-wide absence.

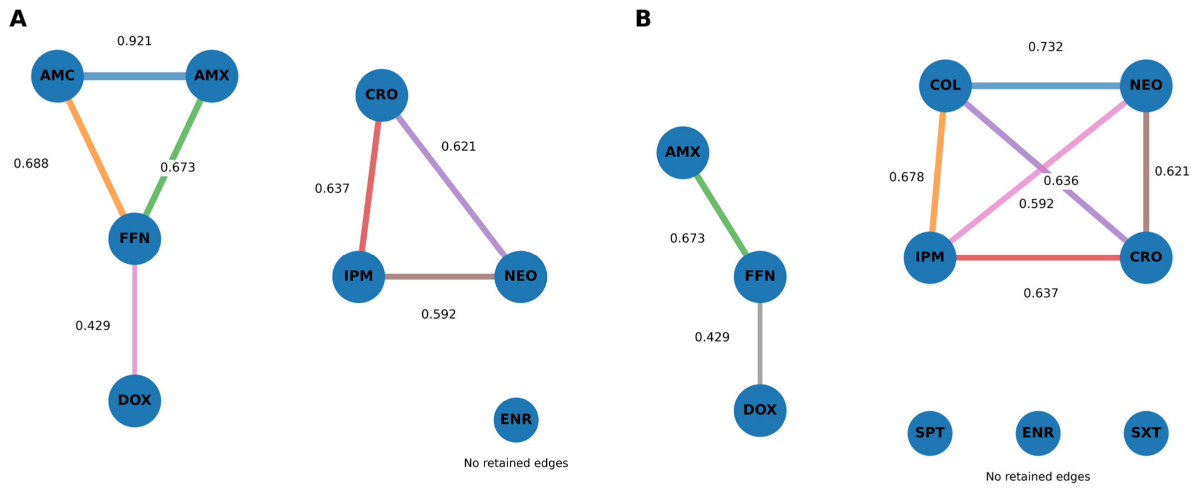

Supplementary Figure S1. Sensitivity analyses of the *Escherichia coli* MIC-covariation network. (A) Network after exclusion of agents with ≥20% of observations at or above the upper assay boundary (spectinomycin, colistin, and trimethoprim–sulfamethoxazole). (B) Network after exclusion of the amoxicillin–clavulanic acid variable. In both analyses, edges indicate $|\varrho| \geq 0.40$ and Benjamini–Hochberg-adjusted $q < 0.05$.
